# Diabetic choriocapillaris flow deficits affect the outer retina and are related to hemoglobin A1c and systolic blood pressure levels

**DOI:** 10.1038/s41598-023-50132-1

**Published:** 2023-12-19

**Authors:** Norihiro Nagai, Yasuaki Mushiga, Yoko Ozawa

**Affiliations:** 1 Department of Ophthalmology, St. Luke’s International Hospital, Tokyo, Japan; 2 Department of Ophthalmology, Keio University School of Medicine, Tokyo, Japan; 3 Department of Clinical Regenerative Medicine, Fujita Medical Innovation Center Tokyo, and Eye Center, Fujita Health University, Haneda Clinic, 1-1-4, Hanedakuko, Ota-Ku, Haneda Innovation City Zone A, Tokyo, 144-0041 Japan

**Keywords:** Retinal diseases, Biomarkers

## Abstract

Patient systemic and ocular data based on optical coherence tomography (OCT) and OCT angiography images were analyzed (n = 45; control and diabetic eyes with or without diabetic retinopathy [DR]; mean age, 49.6 ± 8.1 years). All participants had best-corrected visual acuity < 0.05 in logMAR. The choriocapillaris flow area (CCFA) ratio was lower and the coefficient of variation (CV) of CCFA ratio was higher in diabetic eyes with or without DR than in control eyes. CCFA ratio of DR eyes was lower than that of diabetic eyes without DR. Superficial retinal vessel length density (VLD) was reduced only in DR eyes. CCFA ratio correlated with retinal VLD, photoreceptor outer segment (PROS) length, and retinal pigment epithelium (RPE) volume in the study population; mean PROS decreased in diabetic eyes with or without DR, and RPE volume increased in DR eyes. CCFA ratio < 65.9% and CV of CCFA ratio ≥ 0.140 were more frequently found in diabetic eyes (odds ratio [OR], 13.333; *P* = 0.001), and related to HbA1c ≥ 7.0% (OR, 4.992; 95% confidence interval [CI] 1.164–21.412; *P* = 0.030) or systolic blood pressure ≥ 135 mmHg (OR, 5.572; 95% CI 1.156–26.863; *P* = 0.032). These findings could help understand diabetic pathogenesis in the choriocapillaris and outer retina, and remind clinicians to manage both diabetes and hypertension.

## Introduction

Diabetic retinopathy (DR) is a leading cause of visual impairment worldwide^1^, and can significantly influence the quality of life^2^. Both systemic as well as ocular disease managements are necessary to prevent the visual impairments^3^. However, patients who do not notice gradual visual impairments in their daily lives may neglect the follow-up and managements, and ultimately suffer from irreversible vision loss. This may be partly because there are currently no established methods to clarify the subtle changes before DR is clinically diagnosed, and/or before visual acuity is decreased. Recognizing the gradual progression of retinal pathogenesis before substantial changes occur would be of immense value in terms of revisiting the need for the continuous management of the condition and preventing the severe visual disorders for both clinicians and patients, and understanding the underlying mechanisms.

Number of patients with diabetes has been increasing^4^, and the disease currently affects approximately 10% of all adults globally^5^; the number of prediabetic individuals, who do not meet the diagnostic criteria but are suspected to have diabetes, is also reported to be increasing. DR affects 30–40% of patients with diabetes^1^ and patients with diabetes are more likely to need retinal consultation in general.

Optical coherence tomography (OCT) angiography (OCTA), a minimally invasive technique, is now being widely used in clinical practice and may also be applicable to patients with diabetes with or without DR. In particular, many clinicians focus on the retinal vessels in the inner retinal layer to determine whether a patient has nonperfusion areas and a necessity of retinal photocoagulation treatment. Moreover, inner retinal disorders in diabetes have been reported both experimentally^6–8^ and clinically^9–11^. In contrast, the pathogenic effects of DR on the retinal outer layers are not well understood.

The choriocapillaris is the innermost layer of the choroid, located adjacent to Bruch’s membrane which separates outer retina from the choroid. It consists of fenestrated capillaries that provide oxygen and nutrients to outer retina composed of the retinal pigment epithelium (RPE) and photoreceptors, and remove waste to enable proper RPE functioning.^12,13^ The choriocapillaris has the highest capillary density in the body^12^, and the flow is regulated neurally and myogenically, and not by hypoxia in contrast to the retinal vessels^14^. Nonetheless, given that diabetes cause microangiopathy^15^, choriocapillaris, not only retinal vessels, may be also affected by diabetes which may influence the retinal conditions, in particular, of the outer retina, and the photoreceptors and the RPE, while the changes had not been well documented.

In this study, OCT and OCTA images of the macular area of patients with diabetes who had not yet experienced substantial visual loss, and were thus assumed not to have severe macular changes, were analyzed. The OCTA images were used to evaluate choriocapillaris flow as per the Phansalkar binarization method and to measure the choriocapillaris flow area (CCFA) ratio^16,17^. The variation in the CCFA ratio according to various locations in the analyzed area was represented in terms of the coefficient of variation (CV) of the CCFA ratio^16,17^. The influence of the diabetic choriocapillaris change on the outer retinal layer; the photoreceptors and the RPE, were evaluated in OCT images. Systemic data were also analyzed to assess the impacts of systemic characteristics, such as hypertension, on local choriocapillaris flow. The study may help further clarify the pathogenesis of DR in the choriocapillaris and the outer retinal layer, and also provide a warning to patients with diabetes who have not yet been diagnosed with DR and/or experienced substantial visual loss, as well as a significance of systemic management in diabetes.

## Results

### Participant characteristics

Twelve control eyes, 15 diabetic eyes without DR, and 18 diabetic eyes with DR, all of which had the best-corrected visual acuity (BCVA) of better than logarithm of the minimum angle of resolution (logMAR) 0.05, no high myopia, and no macular diseases, were analyzed (mean patient age, 49.6 ± 8.1 years) (Table 1). There were no significant differences in age among the three groups (*P* = 0.179). While there were differences in mean BCVA among the three groups (*P* = 0.015), the BCVA of all eyes was better than logMAR 0.05 (mean BCVA, − 0.127 ± 0.061), and there was no difference in mean BCVA between diabetic eyes with and without DR (*P* = 0.984). Central retinal thickness did not differ among the three groups (*P* = 0.741).

**Table 1:** Characteristics of the eyes.

	Control (n = 12)	DM with no DR (n = 15)	DR (n = 18)	*P*^a)^	*P*^b)^	*P*^c)^
Age	44.8 ± 12.1 (27–61)	50.0 ± 5.8 (37–60)	52.5 ± 4.9 (42–60)	0.179	0.379	0.198
Sex (male)	2 (16.7%)	8 (53.3%)	15 (83.3%)	0.001**	0.058	0.068
logMAR BCVA	− 0.17 ± 0.03 (− 0.18–0.08)	− 0.11 ± 0.08 (− 0.18–0.05)	− 0.12 ± 0.05 (− 0.18–0.05)	0.015*	0.015*	0.984
Central retinal thickness (μm)	264 ± 17 (236–289)	269 ± 10 (248–285)	264 ± 24 (226–304)	0.741	0.541	0.871

Data are presented as ranges (mean ± standard deviation); ^a)^ Kruskal–Wallis test and ^b, c)^ Mann–Whitney U test. Comparisons between ^b)^ control and diabetic eyes with no DR (DM with no DR), and ^c)^ DM with no DR and DR. DM, diabetes mellitus; DR, diabetic retinopathy; BCVA, best-corrected visual acuity. *P < 0.05, **P < 0.01.

### Choriocapillaris and superficial retinal vascular flow in diabetes

**Figure 1:**
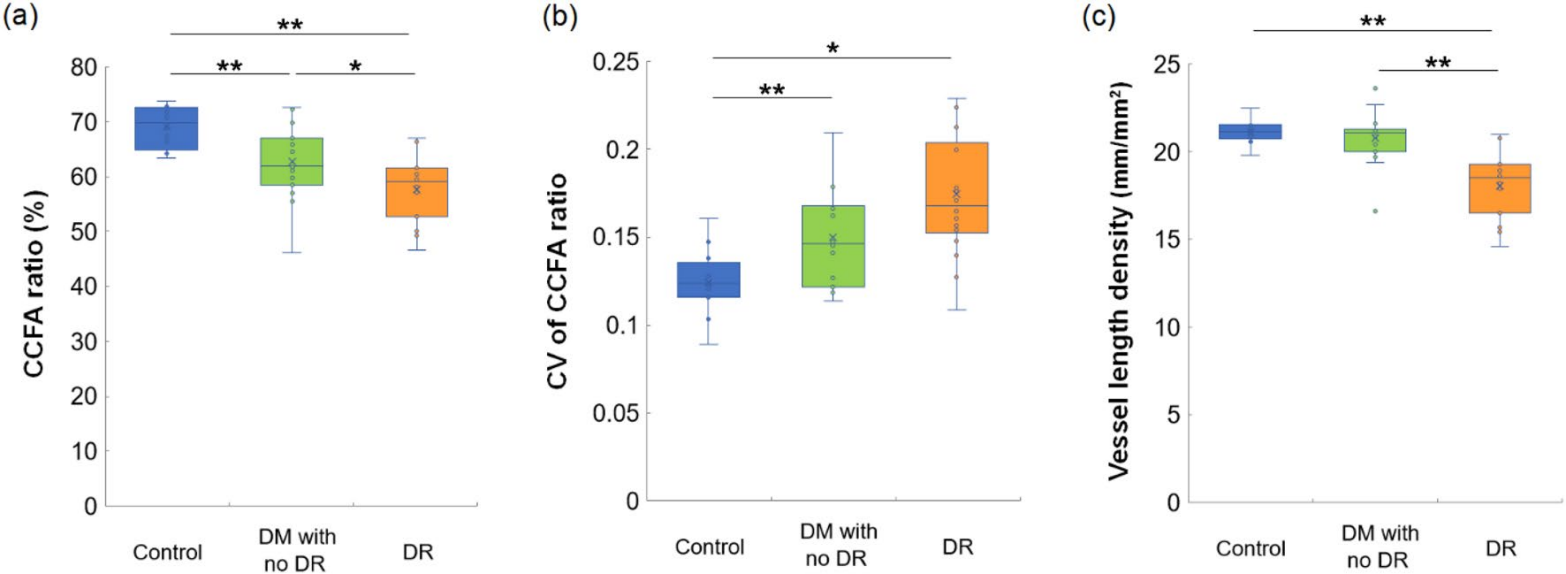
Choriocapillaris flow area (CCFA) ratio, coefficient of variation (CV) of the CCFA ratio, and superficial retinal vessel length density (VLD) of control and diabetic (DM) eyes with or without diabetic retinopathy (DR). Differences among the three groups were noted in terms of the (**a**) CCFA ratio, *P* < 0.001; (**b**) CV of the CCFA ratio, *P* < 0.001; and (**c**) VLD, *P* < 0.001; Kruskal–Wallis test. *P < 0.05 and **P < 0.01; Mann–Whitney U test for comparisons between two groups.

To evaluate deficits and heterogeneity of the choriocapillaris flow, the CCFA ratio of the total analyzed area and the CV of the CCFA ratio that represents variations of the local CCFA ratio were analyzed (Fig. 1a,b). The mean CCFA ratio was significantly lower in both diabetic eyes without DR (62.7 ± 6.9%, *P* = 0.010) and with DR (58.4 ± 6.1%, P < 0.001) than that in control eyes (69.1 ± 3.8%). The mean CCFA ratio of diabetic eyes with DR was lower than that of diabetic eyes without DR (*P* = 0.027). The mean CV of the CCFA ratio of both diabetic eyes without DR (0.150 ± 0.028, *P* = 0.028) and diabetic eyes with DR (0.174 ± 0.035, *P* < 0.001) was higher than that of control eyes (0.124 ± 0.019). Thus, choriocapillaris flow was heterogeneously reduced in diabetic eyes.

Retinal vascular flow was evaluated by measuring the mean foveal vessel length density (VLD) in the superficial retinal layer. The mean VLD of eyes with DR (18.2 ± 1.7 mm/mm^2^) was lower than that of control eyes (21.2 ± 0.7 mm/mm^2^, *P* < 0.001) and diabetic eyes without DR (20.7 ± 0.7 mm/mm^2^, *P* < 0.001) (Fig. 1c).

### Correlation between choriocapillaris and superficial retinal vascular flow

**Figure 2:**
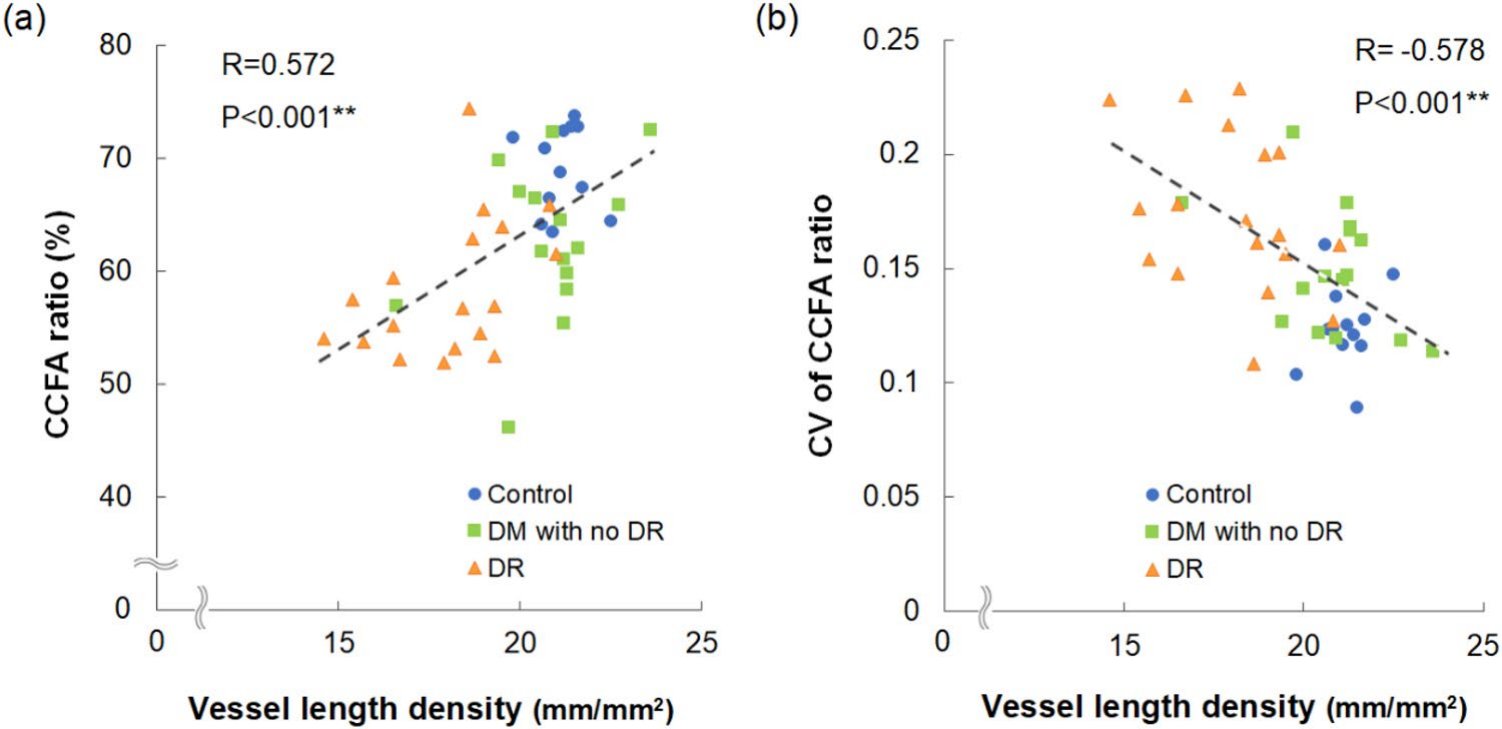
Correlation of superficial retinal vessel length density (VLD) with choriocapillaris flow area (CCFA) ratio and the coefficient of variation (CV) of the CCFA ratio. CCFA ratio (**a**) was positively and the CV of the CCFA ratio (**b**) was negatively correlated with the superficial retinal VLD. **P < 0.01.

The CCFA ratio was positively (R = 0.572, *P* < 0.001) (Fig. 2a) and the CV of the CCFA ratio was negatively (R = − 0.578, *P* < 0.001) (Fig. 2b) correlated with the superficial retinal VLD in the whole study population, indicating that the choriocapillaris and retinal vascular flow were closely related to each other.

### Correlation between choriocapillaris flow and morphology of the retinal outer layer

**Figure 3:**
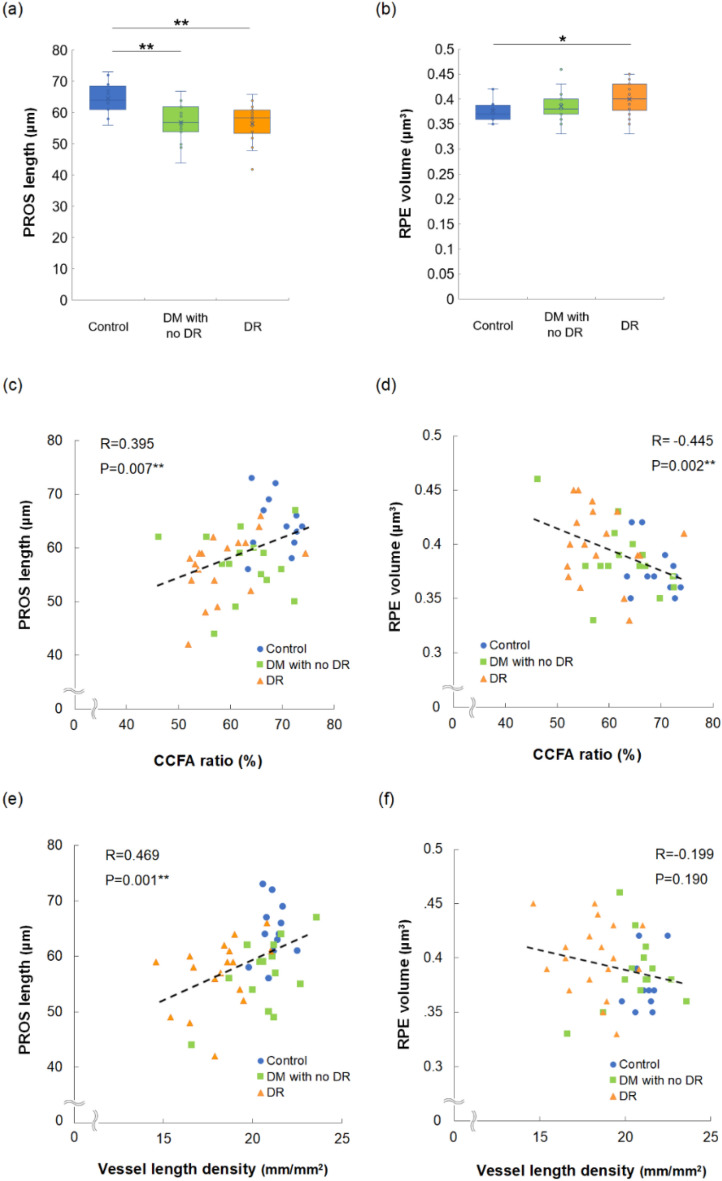
Photoreceptor outer segment (PROS) length and retinal pigment epithelium (RPE) volume of control eyes and diabetic (DM) eyes with or without diabetic retinopathy (DR) and the correlations between the parameters in the study population. Differences were noted among the three groups in terms of PROS length (**a**, *P* = 0.003) and RPE volume (**b**, *P* = 0.065); Kruskal–Wallis test. CCFA was correlated with PROS length (**c**) and RPE volume (**d**), and superficial retinal superficial VLD was correlated with PROS length (**e**) but not with RPE volume (**f**); *P < 0.05 and **P < 0.01, Mann–Whitney U test.

Next, we analyzed the morphologies of the retinal outer layers, photoreceptor layer, and RPE, which the choriocapillaris nourishes, and their associations with choriocapillaris flow. The length of the photoreceptor outer segment (PROS) in diabetic eyes both with (56.7 ± 6.0 μm, *P* = 0.002) and without DR (57.0 ± 6.0 μm, *P* = 0.004) was lower than that in control eyes (64.5 ± 5.2 μm) (Fig. 3a). Moreover, RPE volume in the macular area of diabetic eyes with DR (0.40 ± 0.03 μm^3^) was higher than that in control eyes (0.38 ± 0.02 μm^3^, *P* = 0.033) (Fig. 3b). CCFA ratio was positively correlated with PROS length (R = 0.395, *P* = 0.007) (Fig. 3c) and negatively correlated with RPE volume (R = − 0.445, *P* = 0.002) (Fig. 3d). Collectively, these results indicated that choriocapillaris flow deficits and morphological changes in the retinal outer layer were closely related. Superficial retinal VLD was positively correlated with PROS length (R = 0.469, *P* = 0.001) (Fig. 3e), but not with RPE volume (R = − 0.199, *P* = 0.190) (Fig. 3f).

### Systemic factors affecting choriocapillaris flow

**Figure 4:**
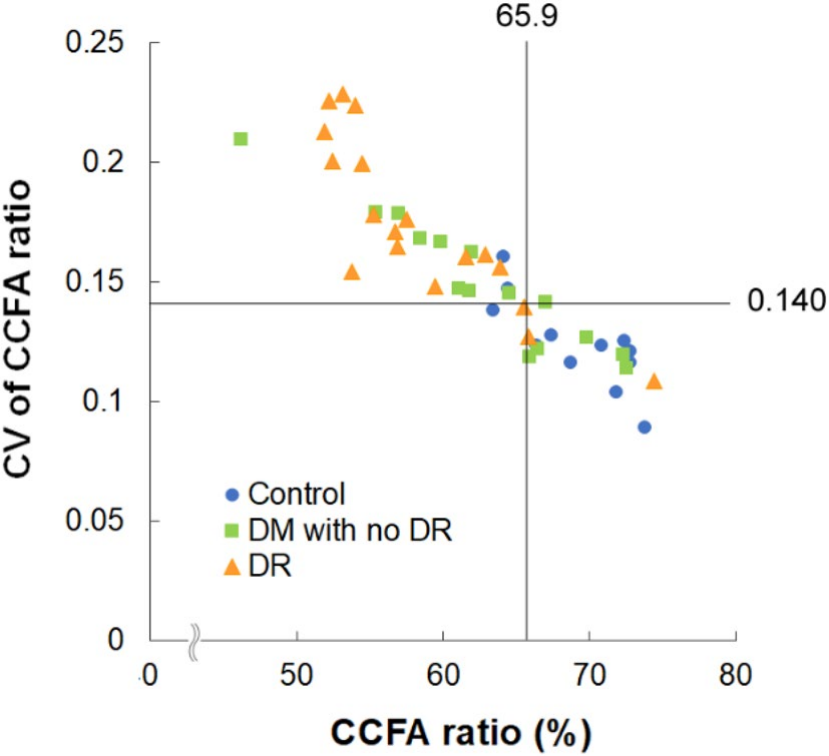
Indices for diabetic choriocapillaris flow deficits. Scatter plots of choriocapillaris flow area (CCFA) ratio and the coefficient of variation (CV) of the CCFA ratio. Eyes with CCFA ratio < 65.9% and CV of the CCFA ratio ≥ 0.140 were found to be at an increased risk of eyes of patients with diabetes (odds ratio, 13.333; *P* = 0.001).

Finally, the systemic factors affecting choriocapillaris flow were analyzed. The detailed blood pressure (BP) and laboratory test result data are presented in Supplementary Table S1. To identify the factors related to the heterogeneous deficits in choriocapillaris flow, we first defined the cutoff values of significant choriocapillaris flow changes in diabetes. According to the receiver operating characteristic area under the curve values (0.859 for the CCFA ratio and 0.838 for the CV of the CCFA ratio; Supplementary Figure S1), eyes with a CCFA ratio < 65.9% and a CV of the CCFA ratio ≥ 0.140 had a higher risk of eyes of patients with diabetes (odds ratio [OR], 13.333; *P* = 0.001) (Fig. 4), and the choriocapillaris flow with a CCFA ratio < 65.9% and a CV of the CCFA ratio ≥ 0.140 was defined as impaired flow in the current study.

We then analyzed the systemic risk factors for diabetic choriocapillaris flow impairment based on the above-mentioned cutoff values. Multiple logistic regression analysis adjusted for age showed that a hemoglobin A 1c (HbA1c) concentration > 7.0 mg/dl was associated with a 4.992-fold higher risk (95% confidence interval [CI] 1.164 to 21.412, *P* = 0.030) and a systolic BP ≥ 135 mmHg was associated with a 5.572-fold higher risk (95% CI 1.156 to 26.863, *P* = 0.032) of deficits and imbalance of choriocapillaris flow in diabetes after adjusting for age (Table 2).

**Table 2:** Systemic risk factors for eyes with heterogenous choriocapillaris flow deficits defined according to the choriocapillaris flow area (CCFA) ratio < 65.9% and the coefficient of variation (CV) of the CCFA ratio ≥ 0.140.

	OR	95%CI	*P*
HbA1c > 7.0 mg/dl	4.992	1.164–21.412	0.030
Systolic BP ≥ 135 mmHg	5.572	1.156–26.863	0.032
Diastolic BP ≥ 85 mmHg	1.207	0.330–4.418	0.776
HDL-C < 40 mg/dl	1.759	0.162–19.069	0.642
LDL-C ≥ 140 mg/dl	1.148	0.264–4.992	0.854
LDL/HDL ratio ≥ 2.5	1.628	0.409–6.480	0.489
Triglyceride ≥ 150 mg/dl	0.946	0.256–3.491	0.934
Total cholesterol ≥ 220 mg/dl	0.689	0.176–2.690	0.592
Non-HDL-C ≥ 170 mg/dl	0.873	0.195–3.907	0.859

Multiple logistic regression analysis adjusted for age. CCFA, choriocapillaris flow area; CV, coefficient of variation. BP, blood pressure; HDL-C, High density lipoprotein-cholesterol; LDL-C, Low density lipoprotein-cholesterol. *P < 0.05.

**Figure 5:**
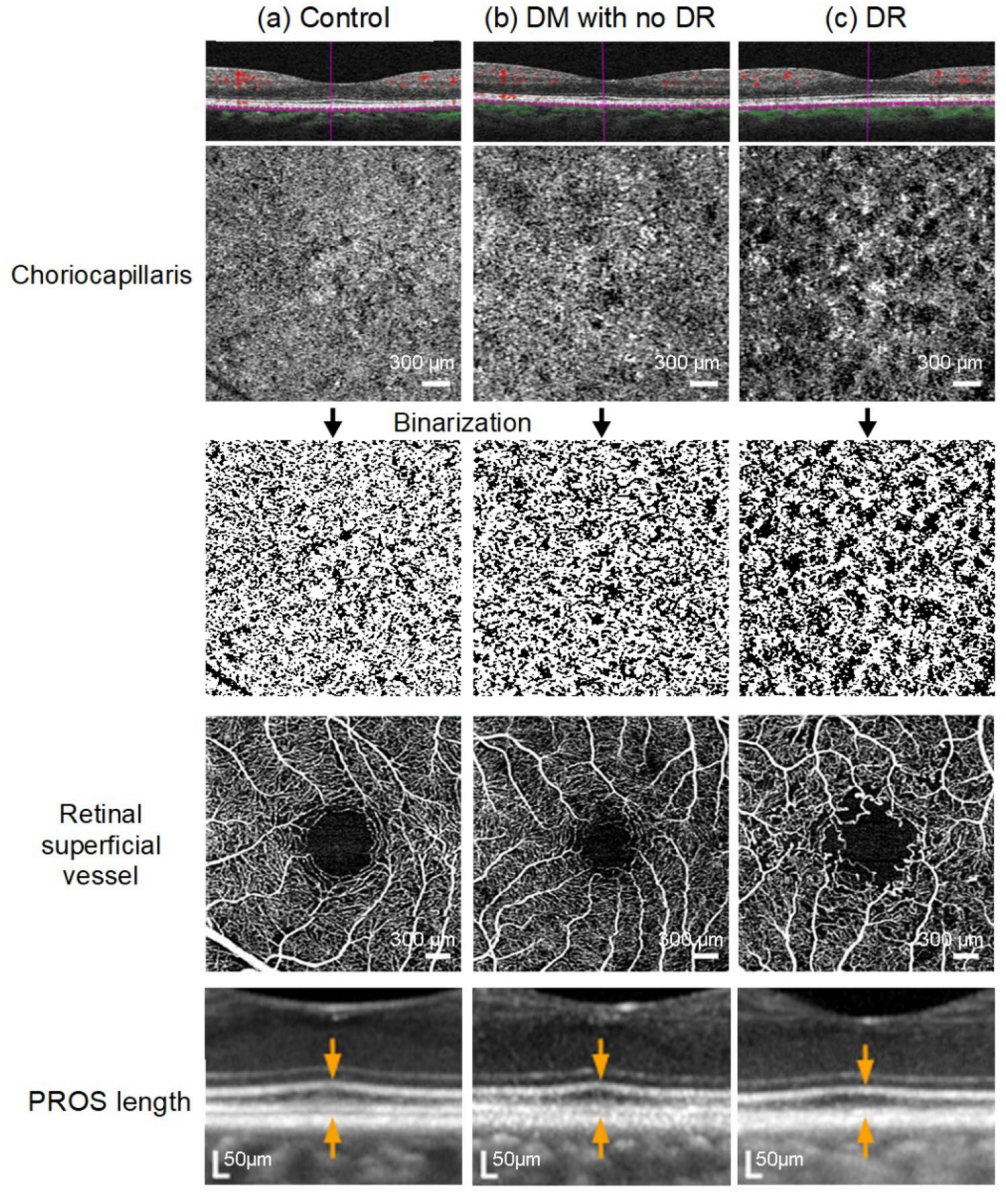
Representative optical coherence tomography (OCT) angiography (OCTA) B-scan and corresponding binarized images of the choriocapillaris slab, OCTA images of the retinal superficial slab, and OCT images of photoreceptor outer segment (PROS) and retinal pigment epithelium (RPE) from control and diabetic (DM) eyes with and without diabetic retinopathy (DR). (**a**) Images of a 61-year-old control participant with a choriocapillaris flow area (CCFA) ratio of 66.4%, coefficient of variation (CV) of the CCFA ratio of 0.123, superficial retinal vessel length density (VLD) of 20.8 mm/mm^2^, PROS length of 67 μm, and RPE volume of 0.42 μm^3^. (**b**) Images of a 50-year-old patient with diabetes without DR, with a CCFA ratio of 61.8%, CV of CCFA ratio of 0.146, superficial retinal VLD of 20.6 mm/mm^2^, PROS length of 59 μm, and RPE volume of 0.43 μm^3^. (**c**) Images of a 54-year-old patient with diabetes and DR with a CCFA ratio of 53.1%, CV of CCFA ratio of 0.229, superficial retinal VLD of 18.2 mm/mm^2^, PROS length 57 μm, and RPE volume of 0.45 μm^3^. Low CCFA ratio represents flow deficits, and high CV of the CCFA ratio represents variation and heterogeneity of the flow according to the location which is observed as local flow voids, while low CV of the CCFA ratio represents relatively homogenous flow in the analyzed area.

Representative OCTA images of the choriocapillaris and retinal superficial vessels and OCT images showing the PROS and RPE are presented in Fig. 5.

## Discussion

The results of this study quantitatively demonstrated that the CCFA ratio was decreased and the CV of the CCFA ratio was increased in the eyes of patients with diabetes with or without DR. The CCFA ratio was further increased in eyes with DR compared with that in eyes of patients with diabetes without DR. Superficial retinal VLD was not affected in the eyes of patients with diabetes and without DR, while it was reduced in eyes with DR. The CCFA ratio was correlated with retinal VLD as well as PROS length and RPE volume in the study population. Finally, the combination of CCFA ratio < 65.9% and CV of the CCFA ratio ≥ 0.140 was associated with increased risk of eyes of patients with diabetes, and was more likely in patients with HbA1c level ≥ 7.0% or systemic BP ≥ 135 mmHg.

Choriocapillaris flow deficits proceeded visible DR and a reduction in superficial retinal VLD, as shown in the current study^18–20^. The deficits progressed in an imbalanced manner—not only was the choriocapillaris flow reduced as a whole, but the flow changes were heterogeneous and spatially random. Choriocapillaris is composed of hexagonal-shaped domains of capillaries; the diameter of the capillary is constant, and diameter of each domain is 200–250 µm^13,21^. The uniform flow in all the domains were shown by experiments using monkeys^22^. Therefore, lower CCFA ratio that show the reduction in the flow, and higher CV of the CCFA ratio that show the non-uniform and heterogenous flow, most likely represent flow impairment. According to one post-mortem study, capillary dropout in the choriocapillaris of patients with diabetes is due to an increased number of polymorphonuclear leukocytes in the vascular lumen^23^. Heterogeneity of the choriocapillaris flow were similar to the changes which we reported in the fellow eyes of patients with unilateral age-related macular degeneration^16,17^, wherein low-grade chronic inflammation is known to be involved in the pathogenesis^24,25^. Diabetes is a chronic inflammatory disease^26^, and the heterogeneous progression of flow deficits may most likely reflect disease progression in response to systemic inflammatory conditions and not to focal stimuli.

The values of choriocapillaris and retinal vascular flow in the study subjects were correlated as a whole, although choroidal flow is regulated by the autonomic nervous system^14^ unlike retinal flow which is autoregulated in response to hypoxia^27^. Whether the correlation is because choroidal and retinal vascular flows come through a common artery, and the ophthalmic artery, would be assessed in the future.

Retinal vessels feed inner retinal layers, and diabetic inner retinal dysfunction has traditionally been assessed using electroretinography^28,29^. However, the outer retinal disorders were also present in the diabetic eyes as shown in the current study. This may be because choriocapillaris flow deficits have influenced photoreceptor health. The studied eyes showed no diabetic macular edema which may cause severe disruptions of ellipsoid zone and external limiting membrane in OCT images and substantial visual loss^30^, but there was a finding in PROS that shows photoreceptor damage. The PROS, where visual pigments responsible for light perception are concentrated, was shortened in diabetic eyes with and even without DR. As a reduction in PROS length is reportedly related to reduction in visual pigment and visual function in animals^31–35^, a subtle reduction in visual quality may have existed in the diabetic eyes, even in the eyes without visible DR, and the BCVA of better than 0.05 in logMAR (0.9 in decimal score).

The RPE volume was increased in eyes with DR where there were choriocapillaris flow deficits. Given that the RPE plays a role in the lipid metabolism required for daily PROS turnover, increase in RPE volume may have been related to accumulation of abnormal lipids^36^, and the RPE disorders.

Taken together, our findings indicated that choriocapillaris flow deficits and imbalances were related to pathological changes in the PROS and RPE, as discussed above, and may be of value for understanding the pathogenesis of DR. The measurement of the PROS and RPE volume may be informative for evaluating early retinal changes in eyes with diabetes, and these changes may explain the decreased quality of vision in patients with diabetes, although further studies are required to validate these aspects.

The cut-off values for choriocapillaris flow impairment in the current study were similar to our previous papers comparing eyes of control healthy patients and fellow eyes of unilateral age-related macular degeneration (AMD); the eyes with CCFA ratio < 60.0% and CV of the CCFA ratio ≥ 0.154 had an AMD risk^16^. So, these parameters would be of value to estimate the disease risk. However, further studies with more participants would be required to define exact values to represent normal range of flow. This would be a future research interest.

Choriocapillaris changes were associated with HbA1c levels and systolic hypertension after adjusting for age. HbA1c level < 7% is the therapeutic target when aiming to prevent complications according to the Japanese guidelines for diabetes^3^. In fact, maintaining the HbA1c level at < 7% over the 5-year follow-up period has been reported to be associated with reduced odds of diabetes-related cardiovascular complications^37^. Blood sugar levels may directly affect the induction of oxidative stress in vascular endothelial cells^38^ and/or affect leukocytes to influence endothelial cells, thus affecting choriocapillaris flow. Alternatively, oxidative stress and leukocyte attack can also affect photoreceptors and the RPE, which may have affected choriocapillaris flow, as the RPE physiologically provides vascular endothelial growth factor to the choriocapillaris to control blood flow^39^.

Systolic BP ≥ 135 mmHg was also found to be a risk factor for diabetic choriocapillaris flow deficits in this study. This is consistent with previous reports that these deficits are related to a poorly controlled hypertensive condition and renal dysfunction, another phenotype of microangiopathy^40^, and hypertensive retinopathy as defined by the Keith-Wagener-Barker classification^41^. Strict BP control proved valuable for suppressing the worsening of the retinal condition and visual function in patients with type 2 diabetes and hypertension in a large-scale clinical study (the United Kingdom Prospective Diabetes Study). Whether lowering blood sugar levels and treating hypertension in patients with diabetes preserves and/or improves choriocapillaris flow after impairment has occurred will be studied in the future.

The limitations of the current study included its relatively small sample size, with different numbers of the subjects in each group, and cross-sectional design. The groups were not divided by the grade of DR. VLD was automatically measured by built-in software in the OCT device and the measurement area was not corrected by axial length. However, this study provides potential value for assessing diabetic choroidopathy, particularly choroidal microangiopathy in the choriocapillaris, which may affect the outer retinal layers. Also, the results may encourage the future analyses of large-scale clinical data to establish a biomarker with defined cutoff values for CCFA ratio and CV of the CCFA ratio.

In conclusion, the results of this study indicated that choriocapillaris flow deficits progress heterogeneously with diabetes and may precede superficial retinal vascular flow deficits and clinically diagnosed DR. Choriocapillaris changes correlated with PROS shortening and RPE thickening, which could theoretically influence visual function. High HbA1c levels and hypertension were found to be risk factors for diabetic choriocapillaris flow deficits. Thus, evaluation of the choriocapillaris may be valuable for understanding the pathogenesis of visual quality disorders in eyes with diabetes. In particular, choriocapillaris flow impairment which most likely affected outer neural retina could be a new perspective for evaluating the influence of diabetes on the visual system. Also, not only management of diabetes, but of hypertension would be of value to control the visual conditions. Accordingly, further studies on whether choriocapillaris changes may be biomarkers for very early changes in visual system in diabetes, and DR progression would be warranted.

## Methods

### Participants

Fifteen eyes of 15 patients with diabetes but no DR, 18 eyes of 18 patients with diabetes and DR, including 10 eyes with non-preproliferative DR and 8 eyes with proliferative DR, and 12 eyes of 12 healthy, age-matched participants were analyzed in this study. Subjects with diabetes were recruited from the out patients, and subjects for control were from those who applied for the study after seeing the announcement poster for the current study in the hospital according to the instruction by the Ethics Committee, and were included if they met all of the following criteria: (1) BCVA > 0.9 in decimal score (better than logMAR 0.05), (2) axial length < 27 mm, and (3) absence of macular diseases, including macular edema or epiretinal membrane with or without a history of treating macular lesions by anti-vascular endothelial growth factor treatment, or photocoagulation, or surgery. If both of the eyes met the inclusion criteria, we chose the eye with higher CCFA ratio. There were no patients who had systemic vascular diseases, as well as hypertensive retinopathy worse than Kieth Wagner Grade II, retinal vein occlusion, other retinal vascular diseases than DR, and glaucoma. This study adhered to the principles of the Declaration of Helsinki. The St. Luke’s International University Ethics Committee approved this retrospective study (approval number: 20-RK058). Written informed consent for the use of their data for research purposes had been obtained from all participants.

### Eye examinations

BCVA with refraction was evaluated using the Landolt C-chart, and decimal BCVA values were converted to logMAR values. Axial length was measured using an IOLMaster 500 (Carl Zeiss Meditec AG, Jena, Germany). After pupil dilation using 0.5% tropicamide, slit-lamp examination and binocular indirect ophthalmoscopy were performed.

### OCTA

OCTA was used to assess choriocapillaris and retinal vascular flow using a spectral-domain OCT system (CIRRUS 5000; Carl Zeiss Meditec, AG). Images of a 3 × 3 mm pattern were assessed after excluding projection artifacts using the built-in software; only images with a quality index value > 7 were analyzed.

For OCTA image of the choriocapillaris slab, the magnification effect of the OCTA images related to axial length was corrected using the Littmann-Bennett formula^42^. After correction, the size of the analyzed images of the choriocapillaris slab was 2.7 × 2.7 mm (Supplementary Fig. 2a). The flow signal areas in the choriocapillaris slab (CCFAs) were evaluated after binarization based on the Phansalkar local binarization threshold as reported previously^16,17,43^. The CCFAs were calculated using ImageJ (National Institutes of Health, Bethesda, MD, USA; available at http://rsb.info.nih.gov/ij/index.html)^44^. The CCFA ratio was defined as the ratio between the size of the choriocapillaris flow signal area and the size of the analyzed area.

The CV of the CCFA ratio was evaluated to assess the homogeneity or heterogeneity of choriocapillaris flow as we have previously reported^16,17^. The binarized images were split into 18 × 18 smaller images, and the CCFA ratio of each small image was calculated to evaluate the standard deviation and then divided by average.

For VLD measurement, the built-in device software (AngioPlex) was used to automatically measure retinal VLD in the superficial capillary plexus (Supplementary Fig. 2b). VLD represents the total length of the perfused vasculature per unit area^45^.

### OCT

A spectral-domain OCT system (Spectralis OCT; Heidelberg Engineering GmbH, Dossenheim, Germany) was used to analyze retinal thickness and macular volume. PROS length was defined as the distance between the inner border of the ellipsoid zone and the inner border of the RPE, and measured in a single horizontal cross-sectional OCT image of foveal scan, using built-in caliper of the device (Supplementary Fig. 2c). The macular volume of the RPE layer was analyzed using three-dimensional OCT images and the built-in device software according to the Early Treatment Diabetic Retinopathy Study (ETDRS) grid of the retinal layers in 6 mm diameter areas.

### Measurement of BP and blood sample parameters

Systolic and diastolic BP were measured at the arm using an automatic sphygmomanometer. Blood samples were collected, and the concentrations of HbA1c, high-density lipoprotein-cholesterol, low-density lipoprotein-cholesterol, triglycerides, and total cholesterol were analyzed.

### Statistical analyses

IBM SPSS Statistics (version 24.0; IBM Corp., Armonk, NY) was used for all statistical analyses. The Kruskal–Wallis test, Mann–Whitney U test, Pearson’s correlation coefficient analysis, and multiple logistic regression was used to analyze the quantitative data. *P* values < 0.05 were considered statistically significant. All data are presented as the mean ± standard deviation values.

### Ethics declarations

This retrospective study adhered to the tenets of the Declaration of Helsinki, was approved by the St. Luke’s International University Ethics Committee (approval number: 20-RK058), and registered as UMIN000040444.

### Consent to participate/Consent to publish

This retrospective study was approved by the St. Luke’s International University Ethics Committee (approval number: 20-RK058).

### Supplementary Information

Supplementary Information.

## Data Availability

The datasets generated during and/or analyzed during the current study are available from the corresponding author on reasonable request.
