# Supplementary material for: Diabetic choriocapillaris flow deficits affect the outer retina and are related to hemoglobin A1c and systolic blood pressure levels
Source: Sci Rep. 2023 Dec 19;13:22570. doi: 10.1038/s41598-023-50132-1 (PMC10730885; doi:10.1038/s41598-023-50132-1)
Supplement: Supplementary file 1 — Supplementary Information. [file 41598_2023_50132_MOESM1_ESM.docx]

**Diabetic choriocapillaris flow deficits affect the outer retina and are related to hemoglobin A1c and systolic blood pressure levels**

Norihiro Nagai^1,2^, Yasuaki Mushiga^1,2^, and Yoko Ozawa^1,2,3^*

^1^Department of Ophthalmology, St. Luke’s International Hospital

^2^Department of Ophthalmology, Keio University School of Medicine

^3^Department of Clinical Regenerative Medicine, Fujita Medical Innovation Center Tokyo, and Eye Center, Fujita Health University, Haneda Clinic

Running head: Choriocapillaris flow deficits in diabetes

*Correspondence author

Yoko Ozawa M.D., Ph.D

Professor

Department of Clinical Regenerative Medicine

Fujita Medical Innovation Center Tokyo

Eye Center, Fujita Health University, Haneda Clinic

1-1-4, Hanedakuko, Ota-ku,

Haneda Innovation City Zone A,

Tokyo 144-0041

Tel; +81-3-5708-7830

ozawa@a5.keio.jp, yoko.ozawa@fujita-hu.ac.jp

ORCID: 0000-0003-4797-5705ORCID: 0000-0003-4797-5705

**Keywords:** diabetic retinopathy, choriocapillaris, vessel density, photoreceptor, retinal pigment epithelium, blood pressure, diabetic choroidopathy

| Supplementary table 1. Blood pressure and laboratory data | | | | | | |
| --- | --- | --- | --- | --- | --- | --- |
|  | | | | | | |
|  | Control  (n=12) | DM with no DR (n=15) | DR  (n=18) | *P*^a)^ | *P*^b)^ | *P*^c)^ |
| Systolic BP (mmHg) | 123.0 ± 13.3  (104 - 152) | 132.5 ± 22.2 (109 - 199) | 146.7 ± 34.8  (106 - 232) | 0.064 | 0.213 | 0.225 |
| Diastolic BP (mmHg) | 79.6 ± 10.1 (57 - 92) | 80.6 ± 13.9 (62 - 116) | 89.4 ± 20.2  (60 - 139) | 0.203 | 0.826 | 0.181 |
| HbA1c (%) | 5.1 ± 0.3  (4.7 - 5.5) | 7.6 ± 2.2  (5.5 - 14.5) | 8.4 ± 2.1  (6.0 - 12.9) | 0.000** | 0.000** | 0.153 |
| HDL-C (mg/dl) | 74.7 ± 18.1 (51 - 98) | 54.7 ± 13.5 (36 - 77) | 50.7 ± 14.1  (31 - 93) | 0.002** | 0.009** | 0.375 |
| LDL-C (mg/dl) | 125.6 ± 32.1  (75 - 189) | 115.5 ± 53.5  (40 - 215) | 120.6 ± 34.8  (64 - 200) | 0.720 | 0.407 | 0.613 |
| LDL/HDL ratio ≥ 2.5 | 1.80 ± 0.74 (0.94 - 3.50) | 2.28 ± 1.23 (0.70 - 5.10) | 2.58 ± 1.23 (1.02 - 5.97) | 0.160 | 0.354 | 0.588 |
| TG (mg/dl) | 91.0 ± 45.2  (32 - 161) | 224.3 ± 170.2  (71 - 759) | 258.3 ± 202.5  (70 - 847) | 0.001** | 0.002** | 0.718 |
| T-C (mg/dl) | 211.3 ± 34.0  (160 - 259) | 193.3 ± 54.5  (118- 293) | 210.9 ± 59.1  (133 - 386) | 0.711 | 0.479 | 0.772 |
| Non-HDL-C (mg/dl) | 136.7 ± 34.7 (80 - 200) | 147.7 ± 56.9 (61 - 236) | 160.2 ± 62.4 (93 - 347) | 0.779 | 0.733 | 0.885 |
| Data are presented as ranges (mean ± standard deviation); ^a)^ Kruskal-Wallis test and ^b, c)^ Mann–Whitney U test. Comparisons between ^b)^ control and diabetic eyes with no DR (DM with no DR), and ^c^) DM with no DR and DR. DM, diabetes mellitus; DR, diabetic retinopathy; BP, blood pressure; HbA1c, Hemoglobin A 1c; HDL-C, High density lipoprotein-cholesterol; LDL-C, Low density lipoprotein-cholesterol; T-C, total cholesterol; Non-HDL-C, non-High density lipoprotein-cholesterol. ***P*<0.01. | | | | | | |

**
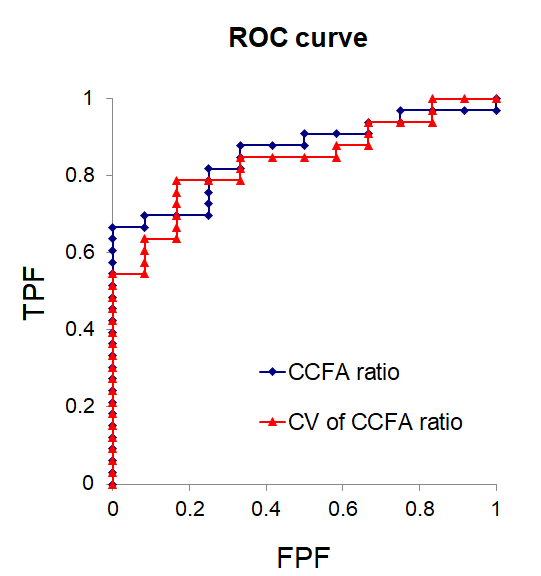
**

**Supplementary Figure 1. ROC curve for CCFA ratio and CV of the CCFA ratio.**


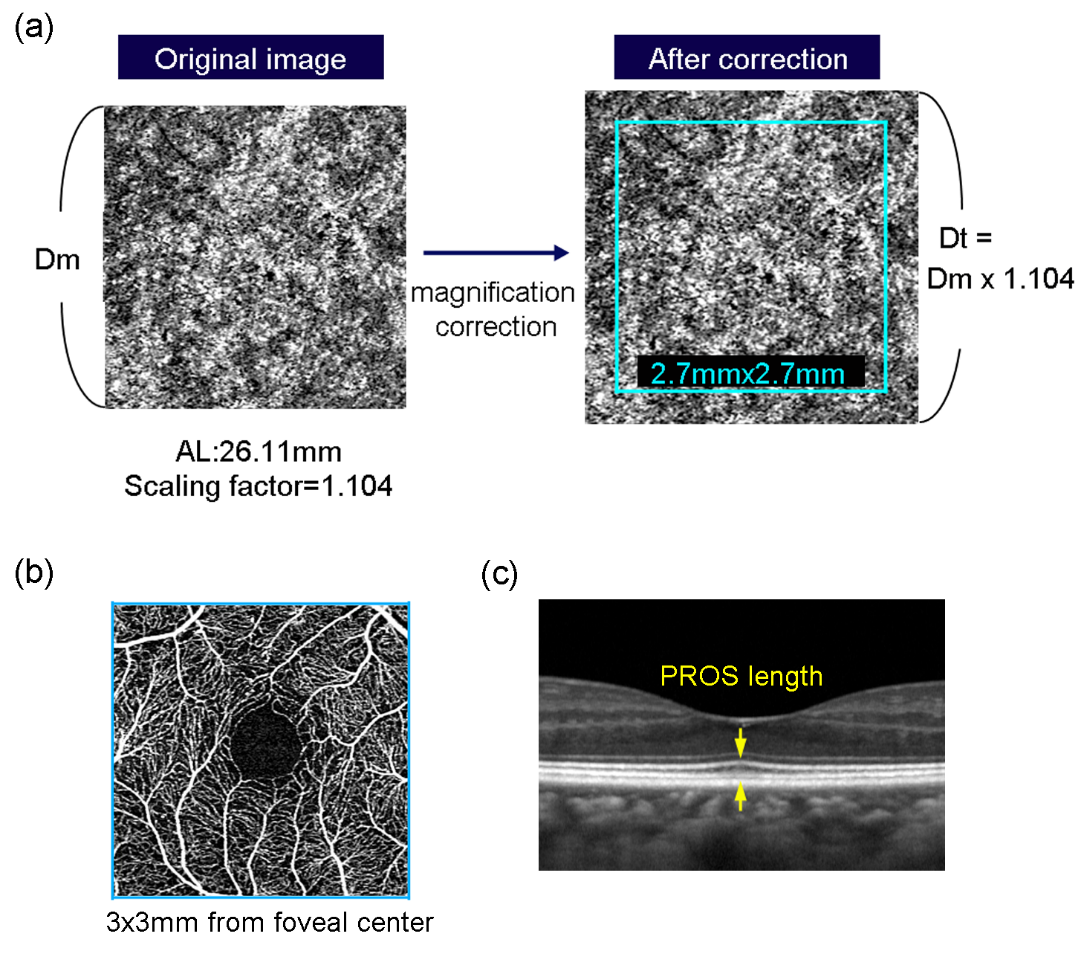


**Supplementary Figure2. Measurement of choriocapillaris flow area (CCFA), vascular length density (VLD), and photoreceptor outer segment (PROS) length**

(a) Magnification correction in the OCTA image of choriocapillaris slab was performed referring to axial length (AL). Scaling factor = 3.382 × 0.013062 × (AL − 1.82) (Littmann’s method and the Bennett formula). The scaling factor shows the magnification effect and the division of the true diameter (Dt) by measured diameter (Dm) (Dt /Dm). If the eye has AL of 26.11mm, the Dt /Dm is 1.104, and the measured area corresponds to 3.11x3.11mm in true diameters. Meanwhile, if the eye has short axial length, the Dt /Dm may be smaller than 1, and the true diameter may become less than 3mm. Considering the smallest true diameter in the current study, we used 2.7x2.7mm true area of the corrected images from all the participants after binarization. Then, choriocapillaris flow area was measured using ImageJ (National Institutes of Health, Bethesda, MD, USA; available at http://rsb.info.nih.gov/ij/index.html).

(b) Measurement of vascular length density (VLD) in the superficial retinal layer. The VLD was measured using built-in device software (AngioPlex) in the 3x3mm OCTA images of superficial capillary plexus, automatically.

(c) Measurement of photoreceptor outer segment (PROS) length. A single horizontal cross-sectional OCT image of foveal scan was magnified 400x and PROS length was measured using built-in caliper, manually, by a retina specialist (NN). The PROS length was defined as the distance between the inner border of the retinal pigment epithelium and the inner border of the ellipsoid zone at the fovea (between yellow arrows).
